# Supplementary material for: Effectiveness of Naldemedine Compared with Magnesium Oxide in Preventing Opioid-Induced Constipation: A Randomized Controlled Trial
Source: Cancers (Basel). 2022 Apr 24;14(9):2112. doi: 10.3390/cancers14092112 (PMC9102438; doi:10.3390/cancers14092112)
Supplement: Supplementary file 1 [file cancers-14-02112-s001.zip › cancers-1688585-supplementary.pdf]

Table of Contents

Supplementary Table S1. Patient inclusion and exclusion criteria....................2
Supplementary Table S2. Study endpoints....................3
Study protocol....................4-29

Supplementary Table S1. Patient inclusion and exclusion criteria

Inclusion and exclusion criteria
Description of inclusion criteria
Males and females; 20–85 years of age
Patients who have not started opioid therapy
Patients who will commence opioid therapy for cancer pain
Patients capable of oral intake
Patients capable of reporting the patient reported outcomes (PRO)
Patients who are prospected to stay in the stable pathological condition during the observation period
Patients who are able to provide written consent to participate in this research, follow instructions during participation, and undergo protocol-specified physical examinations and other examinations, and report their symptoms or events
Description of exclusion criteria
Patients with any contraindications listed on the package insert for magnesium oxide/naldemedine or with a history of hypersensitivity to any ingredients of them
Patients with a serious gastrointestinal structural anomaly (e.g: mechanical ileus), a disease that influences intestinal transit (e.g: paralytic ileus, peritoneal dissemination, peritoneal cancer, uncontrolled hyper/hypothyroidism), irritable bowel syndrome (IBS), inflammatory bowel disease (IBD, e.g: ulcerative colitis, crohn's disease), active diverticular disease, pelvic disorders that cause constipation (e.g: uterine prolapse, rectal prolapse, myoma of the uterus that influences defecation). Or, patients decided by the doctor to have serious influence on gastrointestinal function (e.g: difficulty with oral intake), even if the disease written above are cured
Breastfeeding women or women with possible pregnancy
Patients who had undergone a surgery or a treatment that influences gastrointestinal function (e.g: nerve block) 28days within the enrolment day, or patients planning to take them during the observation period

Supplementary Table S2. Study endpoints

Study endpoints
**Primary endpoint**
・Change in JPAC-QOL from baseline at 2 weeks
**Secondary endpoints**
・Change in SBMs from baseline at 2 and 12 weeks
・Change in JPAC-QOL from baseline at 12 weeks
・Change in PAC-SYM from baseline at 2 and 12 weeks
・Change in CSS from baseline at 2 and 12 weeks
・Change in RomeIV from baseline at 2 and 12 weeks
・Change in BSFS from baseline at 2 and 12 weeks
・Change in SF-36 from baseline at 2 and 12 weeks
**Post-hoc analysis**
・Change in complete spontaneous bowel movement (CSBM)
・JPAC-QOL subscale and JPAC-SYM subscale
・Numerical rating score (NRS) for pain

Abbreviations: JPAC-QOL, Japanese version of Patient Assessment of Constipation Quality of Life; SBM, spontaneous bowel movement; PAC-SYM, Patient Assessment of Constipation-Symptoms; CSS, constipation scoring system; BSFS, Bristol Stool Form Scale; SF-36, short form-36; CSBM, complete spontaneous bowel movement.

Comparing the effectiveness of magnesium oxide and naldemedine in preventing opioid-induced constipation: A proof of concept, single institutional, two arm, open-label, phase II, randomized controlled trial

CLINICAL STUDY PROTOCOL

Takaomi Kessoku, Assistant Professor, Department of Gastroenterology and Hepatology
Yokohama City University Hospital

Ver 1.0

Date of preparation: November 26, 2017

TABLE OF CONTENTS

0. Summery 5
0.1 Study flowchart 5
0.2 Study objectives 5
0.3 Eligibility 5
0.4 Planned number of subjects and study period 6
0.5 Study method 6
0.6 **Principal Investigator** ....
7
1. **Objectives and background of the study** 7
2. **Scientific rationale for the study** 7
2.1 Study Diseases 8
2.2 Current state of treatment 8
2.3 Study Method/Design 8
3. **Study drugs** 8
4. **Diagnostic criteria of the disease** 8
5. **Eligibility** 9
5.1 Inclusion Criteria 9
5.2 Exclusion Criteria 9
6. Patients Registration and Enrollment procedures 9
6.1 Study registration 9
6.2 Registration of other study sites 10
6.3 Enrollment Procedures 10
6.4 Method of randomization 10
6.5 Blinding of patients and physicians 10
7. **Study treatment** 10
7.1 Name of study drugs 10
7.2 Criteria for dose reduction and drug withdrawal 11
7.3 Criteria for discontinuation 11
7.4 Concomitant Therapy 11
7.4.1 Prohibited Concomitant Therapy 11
7.4.2 Continuous Prior Treatment and Concomitant Therapy 11
7.5 After the study 12
8. Summary of Known and Potential Risks and Benefits in Patients 12

8.1 Potential Risks and Benefits in Patients 12
8.2 Definitions of Adverse Events 12
8.3 Assessment of Adverse Events 12
8.4 Expected Adverse Events 13
8.5 Response to Serious Adverse Events (SAE) 14
8.5.1 Emergency Reporting of Serious Adverse Events 14
8.5.2 General Reporting of Adverse Events 14
9. Study schedule 14
9.1 Planned Duration of Patient Participation 14
9.2 Observation, Test and Investigation Items 14
9.2.1 Patient's background 14
9.2.2 Clinical findings 14
9.2.3 Compliance with treatment/ assessment of QOL 15
9.3 Observation, Test and Investigation schedule 15
10. Study endpoints 16
10.1 Primary Endpoint 16
10.2 Secondary Endpoints 16
10.2.1 Efficacy endpoints 16
10.2.2 Safety endpoint 16
10.2.3 Other endpoints 16
11. Statistical Analysis 16
11.1 Planned Sample Size 16
11.2 Statistics Analysis Plan 17
11.3 Interim Analysis 17
12. Case Report Forms 17
13. Efficacy and Safety Evaluation Committee 17
14. Ethical issues 18
14.1 Ethical Conduction of Study 18
14.2 Handling of Personal Information 18
15. Patient Informed Consent and Information Provision 18
15.1 Response to consultation from the participants and their related persons 19
15.2 Obtaining informed consent on behalf of the participant 19
15.3 Obtaining informed assent 19
15.4 Conducting study when subjects are in immediate or obvious danger of death 19
15.5 Handling of incidental finding 19
16. Retention and disposing of Records 20

17. Status of funding and conflicts of interest 20
17.1 Study funds 20
17.2 Expenses related to the study 20
17.3 Payment for study participants 20
17.4 Compensation for Health Damage and Insurance 20
18. Changes to the study protocol 21
19. Report to the hospital director 21
19.1 Report of progress of the study 21
19.2 Study Termination 21
19.3 Study withdrawal/discontinuation 21
20. Retention of Records 22
21. Disclosure of study information 22
22. Monitoring and audit 22
23. Study Implementation Structure 23
23.1 **Principal Investigator 23**
23.2 Study site 23
23.3 Participating sites 23
23.4 Audit 23
23.5 Biostatistics 23
23.6 Patients allocation and data management 24
23.7 Personal Information Manager 24
24. References List 24

0. Summery

0.1 Study flowchart

This study is a proof of concept, single institutional, two arm, open-label, phase II, randomized controlled trial. Study design is as follows.

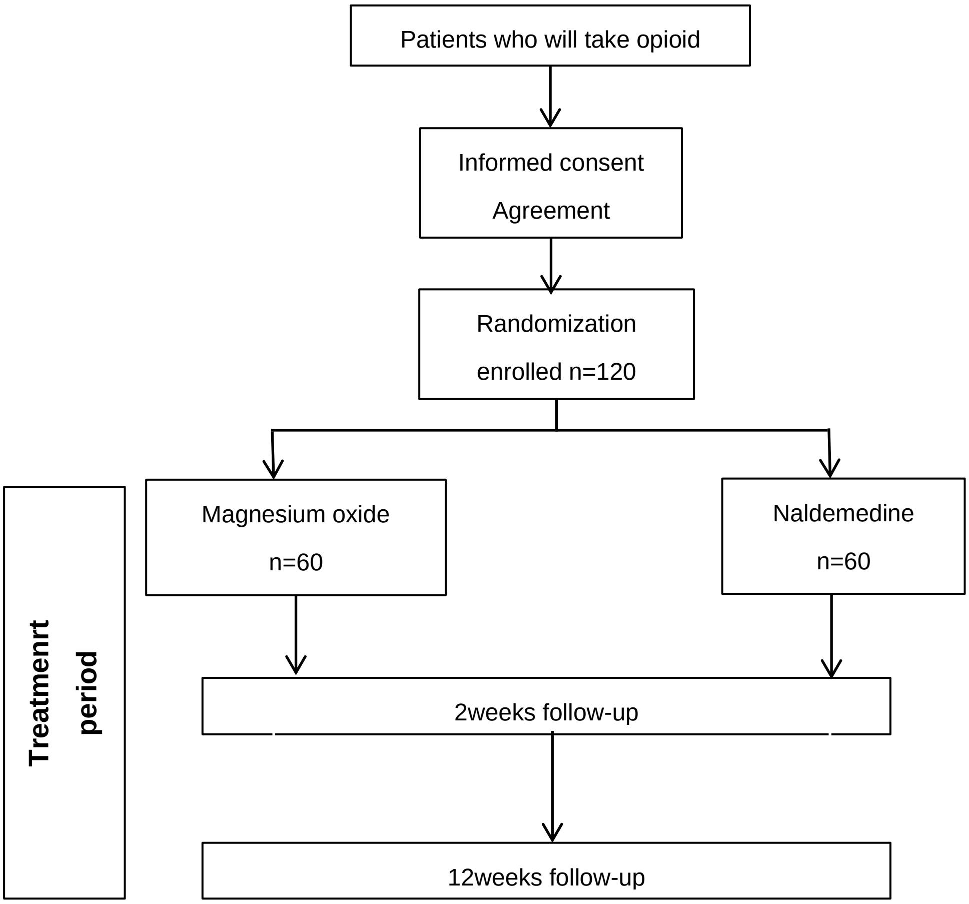

0.2 Study objectives

Opioids are used for cancer pain management but constipation develops in many cases (Opioid-induced constipation; OIC) and impairs patients' quality of life. Thus, OIC is a problem worth investigating. The aim of this study is to compare magnesium oxide to naldemedine and investigate which is more effective in preventing OIC.

0.3 Eligibility

Inclusion Criteria:

1. Patients who received adequate explanation about this study and provided written informed consent
2. Patients who are ≥ 20 and < 85 years of age at the time of informed consent
3. Patients who have not started opioid therapy
4. Patients who are scheduled to commence opioid therapy for cancer pain
5. Patients capable of oral intake
6. Patients capable of reporting the patient reported outcomes (PRO)
7. Patients who are prospected to stay in the stable pathological condition during the observation period

Exclusion Criteria:

1. Patients with any contraindications listed on the package insert for magnesium oxide/naldemedine or with a history of hypersensitivity to any ingredients of these medicine
2. Patients with a serious gastrointestinal structural anomaly (e.g: mechanical ileus), a disease that influences intestinal transit (e.g: paralytic ileus, peritoneal dissemination, peritoneal cancer, uncontrolled hyper/hypothyroidism), irritable bowel syndrome (IBS), inflammatory bowel disease (IBD, e.g: ulcerative colitis, crohn's disease), active diverticular disease, pelvic disorders that cause constipation (e.g: uterine prolapse, rectal prolapse, myoma of the uterus that influences defecation). Or, patients decided by the doctor to have serious influence on gastrointestinal function (e.g: difficulty with oral intake), even if the disease written above are cured
3. Breastfeeding women or women with possible pregnancy
4. Patients who had undergone a surgery or a treatment that influences gastrointestinal function (e.g: nerve block) 28days within the enrolment day, or patients planning to take them during the observation period

0.4 Planned number of subjects and study period

Planned number of subjects: 120 subjects (60 for each group)

Study period: From March 22, 2018 to March 31, 2023

0.5 Study method

The study consists of a screening period for 4 weeks and a treatment period for 12 weeks. Cancer patients scheduled to commence opioid therapy will be randomly

allocated into magnesium oxide (500mg (1 tablet), thrice daily after each meal) or naldemedine (0.2 mg/day (1 tablet), once daily after breakfast) groups and will orally administer each study drug for 12 weeks during the treatment period.

The primary endpoint will be the change in the Japanese version of The Patient Assessment of Constipation Quality of Life (JPAC-QOL) from baseline at 2 weeks. The Patient Assessment of Constipation Symptoms (PAC-SYM), the Constipation Scoring System (CSS), the Bristol Stool Form Scale will also be examined. The presence of constipation will be assessed before and after drug administration using the Rome IV criteria.

0.6 **Principal Investigator**

Takaomi Kessoku
Department of Gastroenterology and Hepatology, Yokohama City University Hospital
Tel：045-787-2640

1. Objectives and background of the study

Opioids are used for cancer pain management [1,2]; however, there are challenges associated with continuous opioid therapy owing to complications such as nausea, constipation, sleepiness, and respiratory depression [3-6]. Constipation develops in 15-64% of patients receiving strong opioid analgesics [7–11]. Long duration of opioid therapy is greatly responsible for opioid-induced constipation (OIC) [12] and drug tolerance against OIC is barely made, so preventive administration of laxatives is important [13].

Over the years, little progress has been made in OIC treatment research [9]. Conventional OIC treatment involves a “non-drug” therapy comprising the consumption of high fiber diets or medications like laxatives, but neither of them targets the underlying mechanism of OIC [3,9]. OIC occurs when opioids act on μ-receptors in the intestinal nerves, reducing intestinal motility and intestinal fluid secretion [6,14]. Recently, peripherally acting μ-opioid receptor antagonists (PAMORAs), such as naldemedine, were shown to treat OIC. Naldemedine is a novel PAMORA being developed for the treatment of OIC without affecting central analgesia [15] and safety and efficacy of it has been reported to be superior to that of placebos [16,17].

Symptoms brought by constipation (abdominal pain, abdominal fullness, loss of appetite) impairs patients’ QOL, hence the need for preventive treatment. This study will compare magnesium oxide to naldemedine and investigate which is more effective in preventing OIC.

2. Scientific rationale for the study

2.1 Study Diseases

New, or worsening, symptoms of constipation when initiating, changing or increasing opioid therapy, that must include 2 or more of the following > 25% of the time;

- Lumpy/hard stools
- Sensation of incomplete evacuation
- Sensation of anorectal obstruction/blockage
- Manual maneuvers to facilitate defecations
- Reduced stool frequency (fewer than 3 spontaneous bowel movements per week)

2.2 Current state of treatment

Current OIC treatments include non-drug and drug therapies. Non-drug therapies include abdominal massage and high-fiber diets. For drug treatment, osmotic laxatives are the first choice according to the Palliative Medicine Society's guidelines in Japan and the use of chloride channel activators (lubiprostone) and stimulant laxatives (e.g. senna) is also recommended [11]. For the prevention of OIC, the Society for Palliative Medicine recommends prophylactic administration of magnesium oxide or naldemedine.

2.3 Study Method/Design

This study is an investigator-initiated, single-center, open-label, two-arm, randomized controlled trial. Cancer patients scheduled to commence opioid therapy will be randomly allocated into magnesium oxide (500mg thrice daily) or naldemedine (0.2mg once daily) groups and take each drug for 12 weeks. The endpoints will be the PAC-QOL, PAC-SYM, CSS, Roma IV and Bristol Stool Form Scale.

3. Study drugs

Naldemedine, Magnesium oxide

4. Diagnostic criteria of the disease

New, or worsening, symptoms of constipation when initiating, changing or increasing opioid therapy, that must include 2 or more of the following > 25% of the time;

- Lumpy/hard stools
- Sensation of incomplete evacuation
- Sensation of anorectal obstruction/blockage
- Manual maneuvers to facilitate defecations

・Reduced stool frequency (fewer than 3 spontaneous bowel movements per week)

5. Eligibility

Patients with cancer who meet all the following inclusion criteria and do not meet any of the exclusion criteria will be included in this study.

5.1 Inclusion Criteria

1. Patients who received adequate explanation about this study and provided written informed consent
2. Patients who are ≥ 20 and < 85 years of age at the time of informed consent
3. Patients who have not started opioid therapy
4. Patients who are scheduled to commence opioid therapy for cancer pain
5. Patients capable of oral intake
6. Patients capable of reporting the patient reported outcomes (PRO)
7. Patients who are prospected to stay in the stable pathological condition during the observation period

5.2 Exclusion Criteria

1. Patients with any contraindications listed on the package insert for magnesium oxide/naldemedine or with a history of hypersensitivity to any ingredients of these medicine
2. Patients with a serious gastrointestinal structural anomaly (e.g: mechanical ileus), a disease that influences intestinal transit (e.g: paralytic ileus, peritoneal dissemination, peritoneal cancer, uncontrolled hyper/hypothyroidism), irritable bowel syndrome (IBS), inflammatory bowel disease (IBD, e.g: ulcerative colitis, crohn's disease), active diverticular disease, pelvic disorders that cause constipation (e.g: uterine prolapse, rectal prolapse, myoma of the uterus that influences defecation). Or, patients decided by the doctor to have serious influence on gastrointestinal function (e.g: difficulty with oral intake), even if the disease written above are cured
3. Breastfeeding women or women with possible pregnancy
4. Patients who had undergone a surgery or a treatment that influences gastrointestinal function (e.g: nerve block) 28days within the enrolment day, or patients planning to take them during the observation period

6. Patients Registration and Enrollment procedures

6.1 Study registration

This study will be registered in the UMIN Clinical Trials Registry (UMIN-CTR) [http://www.umin.ac.jp/ctr/index-j.htm] and will be available to the public. The study will be registered prior to its implementation.

6.2 Registration of other study sites

Not applicable.

6.3 Enrollment Procedures

The Investigator or co-investigators will obtain written informed consent from a potential patient. After obtaining patients' consent, the investigator or others will make sure that the patient meets all the inclusion criteria and does not meet any exclusion criteria and then enroll the patients. The investigator or others will fill out the registration form and notify the data center. The data center will check the patient's eligibility and when eligible, the principal investigator or co-investigator will be notified of the patient ID number and drug name by fax by the data center.

Data center:

Department of Gastroenterology and Hepatology, Yokohama City University Hospital

Tel：045-787-2640

Fax：045-784-3546

reception hours: 10:00 a.m. to 5:00 p.m. weekdays

6.4 Method of randomization

Eligible patients will be randomly assigned (1:1) to receive 500 mg magnesium oxide thrice daily, or 0.2 mg naldemedine once daily at the central registration center. Randomization will be carried out after the patient has signed the informed consent form. To avoid a large bias, we will stratify age (<65, ≥ 65) and gender (male, female) using computer-generated administered procedure, in permuted-block method.

6.5 Blinding of patients and physicians

Masking of patients and physicians is not applicable because this is an open label study, but outcome evaluator will be masked to treatment assignment.

7. Study treatment

7.1 Name of study drugs

・Magnesium oxide group:
magnesium oxide 500mg (1 tablet), thrice daily after each meal
orally administer for 12 weeks

・Naldemedine group:
naldemedine 0.2 mg/day (1 tablet) once daily after breakfast
orally administer for 12 weeks

7.2 Criteria for dose reduction and drug withdrawal

When side effects such as diarrhea occur and the physician judges continuing the treatment would be to the detriment of the patient, the dosage will be reduced, or the drug will be withdrawn.

7.3 Criteria for discontinuation

If the patient meets any of the following criteria, the investigator will discontinue the protocol treatment. The "date of discontinuation" is the date when the physician in charge decides to discontinue, not the date when the event that is the reason for discontinuation occurs. Discontinuation cases in (1) through (3) will continue to be observed as long as possible.

(1) When a grade 3 or more severe AE according to the NCI-CACTE version 4.0 occurs
(2) When oral compliance with the study medication is less than 80%
(3) When the patient requests discontinuation of the study
(4) When continuous medical examination becomes challenging due to patient's relocation, change in hospital or busy schedule, etc.
(5) When the patient turns out to be ineligible for the trial after enrollment
(6) When the physician in charge of the study determines that the continuation of the study treatment is not possible for any other reasons

7.4 Concomitant Therapy

7.4.1 Prohibited Concomitant Therapy

(1) Regular use of laxatives other than protocol drugs
(2) Investigational or test drugs other than protocol drugs

7.4.2 Continuous Prior Treatment and Concomitant Therapy

Drugs used more than 4 weeks prior to this study can be used concomitantly if the dosage

and administration have not been changed at least 4 weeks prior to the start of the study. Diet, exercise therapies and symptomatic treatment for complications and adverse events can also be performed concomitantly. Stimulant laxatives are allowed to be used as a rescue medication for constipation.

7.5 After the study

Laxatives can be continued or changed according to the patient's condition.

8. Summary of Known and Potential Risks and Benefits in Patients

8.1 Potential Risks and Benefits in Patients

Taking the study drug may cause occasional or psychological burden. In addition, the study drug may cause side effects due to drug administration. Side effects and adverse events will be appropriately evaluated by medical examination and blood tests at regular visits during the study period. In addition, investigators will make it known to all participants that they can consult with them at any time to promptly respond to any symptoms of side effects that may arise, even outside of the visits specified in the protocol. There is a reasonable possibility that the study drug will provide benefits such as improvement of clinical symptoms and quality of life that have been caused by OIC. There will be no financial benefit from participation in the study.

8.2 Definitions of Adverse Events

Any unfavorable symptoms or signs (including abnormal laboratory values) occurring within 4 weeks of protocol completion will be reported as an adverse event (AE). AEs may or may not be causally related to the protocol treatment.

8.3 Assessment of Adverse Events

The degree of AE will be assessed according to the Common Terminology Criteria for Adverse Events v4.0 as follows.

Grade 1 (mild)	Asymptomatic or mild symptoms; clinical or diagnostic observations only; intervention not indicated
Grade 2 (moderate)	Minimal, local or non-invasive intervention indicated; limitingage-appropriate instrumental ADL

Grade 3 (severe)	Medically significant but not immediately life-threatening; hospitalization or prolongation of hospitalization indicated; disabling;limiting self-care ADL
Grade 4 (life-threatening)	Life-threatening consequences: urgent intervention indicated
Grade 5 (death)	Death related to AE

Causal relationship with protocol treatment is classified into three categories as follows.

Associated: Causal relationship with protocol treatment is reasonable. Not due to exacerbation of the primary disease, complications, or other treatments.

Can be associated: Causal relationship with protocol treatment is unclear. Can be explained by exacerbation of the primary disease, complications or other treatments, etc.

Not associated: No causal relationship with protocol treatment. Can be clearly explained by exacerbation of the primary disease, complications or other treatments, etc.

Serious AEs are defined as follows.

(1) Causes death
(2) Life-threatening
(3) Requires hospitalization or prolonged hospitalization/recuperation
(4) Causes permanent or significant disability or malfunction
(5) Causes congenital anomaly or congenital defect
(6) Other medically important conditions (serious events that require prevention not to reach 1-4 conditions)

8.4 Expected Adverse Events

The efficacy and safety of the study drugs for patients with OIC or constipation have been confirmed in clinical trials, and the inclusion criteria for suitable patients for administration is established. The study will be conducted with adequate safety monitoring. This is an open-label, randomized controlled trial comparing two groups of study drugs, and there is not enough evidence to show that one is superior to the other. In this study, all medical care during the study period, including study treatment, will be provided as usual medical practice. The medical cost will be covered by insurance. The frequency of visits and examinations in this study will

be the same as in the regular medical practice.

Side effects of the study drugs are as follows.

Magnesium oxide: abdominal pain (1.1%), mild diarrhea (1.1%), mild increase in plasma magnesium levels (9.5%)

Naldemedine: diarrhea (21.9%), abdominal pain (2.2%)

8.5 Response to Serious Adverse Events (SAE)

8.5.1 Emergency Reporting of Serious Adverse Events

When a SAE occurs, the co-investigator will immediately report it to the principal investigator. The principal investigator will immediately report the details to the Hospital Director. The hospital director will obtain opinions from the Ethical Review Committee and take necessary measures in accordance with the procedures of the study institution. When an unpredictable SAE which relationship between the study drug cannot be ruled out occurs, the hospital director will promptly report the event to the Minister of Health, Labor and Welfare and notify the principal investigator or the study site.

8.5.2 General Reporting of Adverse Events

When an AE occurs, the principal investigator or co-investigator will write a Case Report Form and report it to the principal investigator (or study site).

9. Study schedule

9.1 Planned Duration of Patient Participation

Protocol treatment period: 12 weeks

Study period: From March 22, 2018 to March 31, 2023

Recruitment period: From March 22, 2018 to March 31, 2021

9.2 Observation, Test and Investigation Items

9.2.1 Patient’s background

The patient’s background will be examined in the baseline period before enrollment. Data from within 1 month prior to enrollment will be used.

- Height, weight, BMI, age, and sex
- Location of cancer, past history, complications, surgical history
- Current medication and dosage
- Allergy

9.2.2 Clinical findings

Patient’s QOL and clinical symptoms will be investigated using the PAC-QOL/PAC-SYM/CSS questionnaires. The presence of constipation will be assessed using the Rome IV criteria and stool shape will be assessed using the Bristol Stool Form Scale. Patients will be randomized after study enrollment and will be seen at 2 weeks (±7 days), 12 weeks (±14 days) and at the time of discontinuation (~+2 days) and the PAC-QOL/PAC-SYM/CSS/Rome IV/ Bristol Stool Form Scale/SMBs questionnaires will be performed at the visits.

9.2.3 Compliance with treatment/ assessment of QOL

Quality of life will be assessed by filling in the questionnaire at 2 weeks after the start of the study and at the end of the study (at 2 and 12 weeks). Compliance with the study medication will be assessed by asking the patients to return the unused tablets at the last visit (at 12 weeks).

9.3 Observation, Test and Investigation schedule

	**Before enrolment**	**treatment period**					
Week	4weeks before enrolment (baseline)	2	4	6	8	10	12
Patients’ background	◎						
Physical examination	◎	◎	○	○	○	○	◎
Symptoms (adverse events)	◎	◎	○	○	○	○	◎
JPAC-QOL	◎	◎					◎
PAC-SYM	◎	◎					◎
CSS	◎	◎					◎
RomeIV	◎	◎					◎

BSFS	◎	◎					◎
SBMs	◎	◎					◎
SF-36	◎	◎					◎

◎ To be performed

○ When necessary

10. Study endpoints

10.1 Primary Endpoint

The change in JPAC-QOL from baseline at 2 weeks

10.2 Secondary Endpoints

10.2.1 Efficacy endpoints

・Change in SBMs from baseline at 2 and 12 weeks

・Change in JPAC-QOL from baseline at 12 weeks

・Change in PAC-SYM from baseline at 2 and 12 weeks

・Change in CSS from baseline at 2 and 12 weeks

・Change in RomeIV from baseline at 2 and 12 weeks

・Change in BSFS from baseline at 2 and 12 weeks

・Change in SF-36 from baseline at 2 and 12 weeks

10.2.2 Safety endpoint

Incidence of adverse events that occurred from the first day to 28 days after treatment

10.2.3 Other endpoints

▪ Protocol compliance and patients' quality of life will be evaluated using the patient diary.

▪ Change in JPAC-QOL subscale at 2 and 12 weeks

▪ Change in JPAC-SYM subscale at 2 and 12 weeks

▪ Change in complete spontaneous bowel movements (CSBMs)

11. Statistical Analysis

11.1 Planned Sample Size

Our retrospective analysis of magnesium oxide/naldemedine in 10 OIC patients at Yokohama City University Hospital showed a mean JPAC-QOL change of -1.19 in the naldemedine group and -0.76 in the magnesium oxide group. We decided to perform

sample size calculation to attain a relevant number for proper analysis of variance F-test based on these data. Assuming mean changes in the JPAC-QOL score in the naldemedine group and magnesium oxide group to be -1.19 and -0.76, respectively, with a common standard deviation of 0.76, 51 patients are needed in each group to reach 90% statistical power with a two-sided significance level of 5%. To compensate for any dropout, we proposed to increase the sample size to 60 per group. To reach this sample size, a total of 120 patients in the study are needed.

11.2 Statistics Analysis Plan

The "full analysis set" is defined as any subject who receives any amount of the study medication with initial information on the primary endpoint. The full analysis set will be the primary analysis set for efficacy to use an *intention-to-treat* patient population.

For the primary endpoint, one-way analysis of variance across the 2 groups will be performed with calculating the p-value using the Student's t-test. The p-value will be significant at a two-sided significant level of 5%. Both the p-value and confidence intervals will be used to determine whether our results are statistically significant or not. The paired t-test or Wilcoxon signed-rank test will be performed to compare before and after the intervention within each group. The chi-squared test will be used to assess the frequency of AE. The treatment compliance rate will be calculated and compared using Fisher's exact test. The JMP version 11.2.0 software (SAS Institute, Cary, NC, USA) will be used for all statistical analyses.

11.3 Interim Analysis

Not applicable.

12. Case Report Forms

Contents: Collection period

Case registration form: at the time of case registration

Start ~ end of protocol treatment: within 14 days after the end of protocol treatment

At the time of discontinuation: within 1 week of visit

13. Efficacy and Safety Evaluation Committee

The purpose of this committee is to ensure the proper conduct of the study and to reduce the burden on subjects as much as possible. The committee will make decisions whether or not to continue the study from the perspective of safety and efficacy. In the event of death or serious adverse events, the committee will decide whether there is a causal relationship and whether appropriate measures were taken. The committee members in charge will be those who have no conflict of interest with the study and will perform their duties from an objective standpoint.

Effectiveness and Safety Evaluation Committee meeting will be held via email or Skype. When an unknown adverse event occurs, the committee will respond in accordance with the separately established procedures.

14. Ethical issues

14.1 Ethical Conduction of Study

The study will be performed in accordance with the Declaration of Helsinki and the Japanese ethical guidelines for clinical research. The physician in charge will conduct the study in compliance with this protocol. If deviations or changes are made from the protocol in order to avoid immediate danger to the subject or for other unavoidable medical reasons, the reasons will be recorded in the medical record and case report form. If the deviation or change is critical to the continuation of the study, the principal investigator and the Efficacy and Safety Evaluation Committee will consider stopping the study or changing the study plan.

14.2 Handling of Personal Information

The principal investigator or co-investigator will assign an identification code to the patient. The identification code will not contain information that can identify the individual study subject, such as the subject's medical record ID or name. A correspondence table that can identify the subjects from the identification codes will be made to identify or refer to the subjects, but it will not be provided outside the study institution. Even when the results of this study are made public, they will not contain the subjects' personal information.

15. Patient Informed Consent and Information Provision

Before the patient participation, the physician in charge will fully explain the following items using the informed consent form approved by the Ethics Committee of the institution. After confirming that the patient fully understands the contents, consent for

participation will be obtained in writing.

In principle, the items to be explained to the subjects will be as follows (this shall not apply to things approved by the hospital director based on the opinion of the Ethical Review Committee);

1. That the study permission has been obtained.
2. Study period and principal investigator
3. Purpose and significance of the study
4. Method and duration of the study
5. Reasons for selection as study subjects
6. Burden and anticipated risks and benefits
7. That the subject can withdraw the study.
8. That the subject will not be disadvantaged.
9. About other treatment methods
10. Access to materials
11. Handling of personal information
12. Methods of storage and disposal of samples and information
13. Conflict of interest
14. Consultation with study subjects and related parties
15. About expenses
16. Provision of medical care after the study is conducted
17. Handling of incidental findings
18. Compensation for health damage
19. Possibility of future study
20. Access to samples and information by concerned parties
21. Publication of study results
22. Intellectual property rights

15.1 Response to consultation from the participants and their related persons

If there is a consultation from the study subjects or their related persons, they should contact the study site, and the consultation will be handled individually according to the contents of the consultation. If the consultation is about symptoms, the participant himself/herself will consult a doctor.

15.2 Obtaining informed consent on behalf of the participant

Not applicable.

15.3 Obtaining informed assent

Not applicable.

15.4 Conducting study when subjects are in immediate or obvious danger of death

Not applicable.

15.5 Handling of incidental finding

There is a possibility that tests performed in this study may reveal a new disease that has not been pointed out before. In such a case, we will proceed with appropriate examination, diagnosis, and management for necessary treatment as usual.

16 Retention and disposing of Records

The information collected in this study will be stored in a locked cabinet in the Department of Hepatology and Gastroenterology in Yokohama City University Hospital. The data will be stored in a database on PC independent of the network. The data will be stored on a password-protected USB and it will be stored in a locked cabinet in the medical office and will be kept for five years. When disposing of the USB, it will be initialized and the data will be deleted. Paper media will be disposed of with a shredder. The information obtained in this study will be stored for five years.

There is a possibility that the information obtained in this study may be used in another study in the future for related or different purposes. In such a case, the study protocol must be prepared anew, approved by the Ethical Review Committee, and approved by the hospital director.

17. Status of funding and conflicts of interest

17.1 Study funds

This study is granted from research funds of Yokohama City University Hospital. This study is conducted from a medical perspective and does not serve the interests or convenience of any particular company or organization. It does not receive funding from any specific company or organization.

17.2 Expenses related to the study

All medical expenses including inspections, drugs, observation, etc. during the study period are covered by health insurance and thus it will be paid by the study subjects.

17.3 Payment for study participants

Not applicable.

17.4 Compensation for Health Damage and Insurance

If any health damage occurred in patients due to this study, the investigator and others must take necessary measures including treatment. The treatment will be provided by health insurance and the subjects will pay his/her own expenses. Since this study is covered by health insurance, if death or permanent disability level 1 or 2 health impairment occurs due to side effects of the drug, the subject will be eligible to apply for relief benefits under the Relief System for Sufferers from Adverse Drug Reactions. In addition, the tests to be performed in this study will be the same as those to be performed in ordinary medical treatment. For these reasons, this study will not be covered by human research insurance.

18. Changes to the study protocol

The principal investigator will make changes to the study protocol when necessary. Regardless of the severity of the changes, the revised protocol and its revision history (details and reasons for the revisions) will be reported to the hospital director in writing.

(1) In case of major changes

Request the Ethics Committee for deliberation and obtain approval. As a criterion for determining severity, it is necessary to consider that the change relates to the scientific nature of the study and that it increases risk to the subjects. Examples include changes related to inclusion criteria, treatment plan, endpoints, anticipated adverse events, etc.

(2) In case of minor changes

Minor changes require the approval of the principal investigator and a report to the Ethics Committee. The review and approval will be in accordance with the institutional arrangements. Refers to changes in the protocol that are not likely to increase the risk to study subjects and are not related to the primary endpoints of the study.

19. Report to the hospital director

19.1 Report of progress of the study

The principal investigator will report progress of the study to the hospital director once a year by writing the implementation status report.

19.2 Study Termination

After study termination, the principal investigator will report the termination and the summary of study results in writing to the hospital director. When the hospital director has received the report of study termination from the investigator, he/she will immediately submit a document describing the IRB of the termination and the study results based on the report submitted from the investigator.

19.3 Study withdrawal/discontinuation

If the study is discontinued or withdrawn, regardless of the reason, the physician in charge will promptly notify the subjects and will implement appropriate measures and tests to confirm the safety of the subjects. The physician in charge will also notify the hospital director in writing.

(1) When it becomes clear that the study treatment is not expected to be effective or that there are safety issues, or if it is determined that the continuation of the study is no longer meaningful.

(2) When it is judged difficult to complete the study due to significant delays in case enrollment or frequent deviations from the protocol, etc.

20. Retention of Records

The principal investigator will be responsible for keeping all documents and records related to this study (e.g., ethics committee records, consent forms, case report forms, etc.) in a separate locked cabinet or password-protected hard drive at the institution, and maintaining them in strict confidence. Records will be retained for 5 years after study completion and then destroyed by the data center.

21. Disclosure of study information

Promptly after the completion of the study, the researcher will write a paper or make a presentation at a conference. The first author will be the principal investigator and co-authors will be determined by the principal investigator. At the time of publication, the principal investigator will review the content.

22. Monitoring and audit

Monitoring is an investigation to ensure that the study is conducted properly, and to what extent the research is being conducted in accordance with the study protocol, procedures, and related laws and regulations, as well as relevant guidelines, by a management team

designated by the principal investigator.

The management team will do the on-site monitoring and meet the facility person in charge when necessary. Any visit to the facility will be reported in the monitoring report. In principle, the first patient will be monitored continuously throughout the trial and if there is no problem, monitoring will be done for every 10 patients. To confirm that necessary documents are stored properly, on-site monitoring will be performed appropriately and if there are any problems, corrective action will be taken. The result will be recorded in the monitoring report and will be submitted to the principal investigator within two weeks.

Monitoring details are as follows;

1) Implementation system of the study institution
2) Status of subject registration and consent acquisition
3) Validity of subject registration
4) Status of storage of materials related to this study, such as subject information, CRFs, etc.
5) Status of compliance with the study protocol
6) Reliability of the contents of the case report (cross-checking with the original materials)
7) Confirmation of study drug/instrument management
8) Appropriate recording and reporting of adverse events
9) Status of registration and termination of public databases
10) Necessary document management

23. Study Implementation Structure

23.1 Principal Investigator

Takaomi Kessoku
Department of Gastroenterology and Hepatology, Yokohama City University Hospital

23.2 Study site

Takaomi Kessoku
Department of Gastroenterology and Hepatology, Yokohama City University Hospital
〒236-0004 A-467, 3-9 Fukuura Kanazawa-ku Yokohama
Tel: 045-787-2640
Fax: 045-784-3546

23.3 Participating sites

Not applicable.

23.4 Audit

Not applicable.

23.5 Biostatistics

Masataka Taguri
Department of Biostatistics, Yokohama City University Graduate School of Medicine

23.6 Patients allocation and data management

Takayuki Kato
Department of Gastroenterology, International University of Health and Welfare Atami Hospital
〒413-0012　13-1 Higashikaigan-cho, Atami, Shizuoka, Japan

23.7 Personal Information Manager

Takaomi Kessoku
Department of Gastroenterology and Hepatology, Yokohama City University Hospital

24. References List

1 P. Gisondi, A. Conti, G. Galdo, S. Piaserico, C. De Simone, G. Girolomoni. Ustekinumab does not increase body mass index in patients with chronic plaque psoriasis: a prospective cohort study. Br. J. Dermatol. 168 (2013) 1124-1127. doi:10.1111/bjd.12235.

2 A. Caraceni, G. Hanks, S. Kaasa, M.I. Bennett, C. Brunelli, N. Cherny, O. Dale, De F. Conno, M. Fallon, M. Hanna, Haugen, D.F. Use of opioid analgesics in the treatment of cancer pain: evidence-based recommendations from the EAPC. Lancet Oncol. 13 (2012) e58-68. doi:10.1016/S1470-2045(12)70040-2.

3 J.L. Poulsen, C. Brock, A.E. Olesen, M. Nilsson, A.M. Drewes. Evolving paradigms in the treatment of opioid-induced bowel dysfunction. Therap. Adv. Gastroenterol. 8 (2015) 360-372. doi:10.1177/1756283X15589526.

4 B. Morlion, Clemens, K.E., Dunlop, W. Quality of life and healthcare resource in patients receiving opioids for chronic pain: a review of the place of oxycodone/naloxone. Clin. Drug. Investig. 35 (2015) 1-11. doi:10.1007/s40261-014-0254-6.

5 M. Lazzari, M.T. Greco, C. Marcassa, S. Finocchi, C. Caldarulo, Corli, O. Efficacy and tolerability of oral oxycodone and oxycodone/naloxone combination in opioid-naive

cancer patients: a propensity analysis. Drug. Des. Devel. Ther. 9 (2015) 5863-5872. doi:10.2147/DDDT.S92998.

6 Camilleri, M. Opioid-induced constipation: challenges and therapeutic opportunities. Am. J. Gastroenterol. 106 (2011) 835-842. doi:10.1038/ajg.2011.30.

7 S. Wirz, M. Wittmann, M. Schenk, A. Schroeck, N. Schaefer, M. Mueller, J. Standop, N. Kloecker, J. Nadstawek. Gastrointestinal symptoms under opioid therapy: a prospective comparison of oral sustained-release hydromorphone, transdermal fentanyl, and transdermal buprenorphine. Eur. J. Pain 13 (2009) 737–743. doi: 10.1016/j.ejpain.2008.09.005.

8 S.F. Cook, L. Lanza, X. Zhou, C.T. Sweeney, D. Goss, K. Hollis, A.W. Mangel, Fehnel, S.E. Gastrointestinal side effects in chronic opioid users: results from a population-based survey. Aliment Pharmacol Ther 27 (2008) 1224–1232. doi: 10.1111/j.1365-2036.2008.03689.x.

9 T.J. Bell, S.J. Panchal, C. Miaskowski, S.C. Bolge, T. Milanova, Williamson, R. The prevalence, severity, and impact of opioid-induced bowel dysfunction: results of a US and European Patient Survey (PROBE 1). Pain Med. 10 (2009) 35–42. doi: 10.1111/j.1526-4637.2008.00495.x

10 M. Yotoku, A. Nakanishi, M. Kanematsu, N. Sakaguchi, N. Hashimoto, F. Koyama, S. Yamaguchi, K. Ikeda, Konishi, H. and Hirotani, Y. Reduction of opioid side effects by prophylactic measures of palliative care team may result in improved quality of life. J. Palliat. Med. 13 (2010) 401–406, doi: 10.1089/jpm.2009.0355.

11 G. Rosti, A. Gatti, A. Costantini, A.F. Sabato, Zucco, F. Opioid-related bowel dysfunction: prevalence and identification of predictive factors in a large sample of Italian patients on chronic treatment. Eur. Rev. Med. Pharmacol. Sci. 14 (2010) 1045–1050.

12 A.K. Tuteja, J. Biskupiak, G.J. Stoddard, Lipman, A.G. Opioid-induced bowel disorders and narcotic bowel syndrome in patients with chronic non-cancer pain. Newrogastroenterol. Motil. 22 (2010) 424-430. doi: 10.1111/j.1365-2982.2009.01458.x.

13 Clinical Guidelines for Cancer Pain Management Second Edition 2014

14 M. Camilleri, D.A. Drossman, G. Becker, L.R. Webster, A.N. Davies, Mawe, G.M. Emerging treatments in neurogastroenterology: a multidisciplinary working group consensus statement on opioid-induced constipation. Neurogastroenterol. Motil. 26 (2014) 1386-1395. doi:10.1111/nmo.12417.

15 Song. X, Wang. D, Qu. X, Dong. N, Teng. S. A meta-analysis of naldemedine for the treatment of opioid-iduced constipation. Expert Rev Clin Pharmacol. 12 (2019) 121-128. doi: 10.1080/17512433.2019.1570845.

16 K. Fukumura, T. Yokota, Y. Baba, J.C. Arjona Ferreira. Phase 1, Randomized,

double-blind, placebo-controlled studies on the safety, tolerability, and pharmacokinetics of naldemedine in healthy volunteers. Clin. Pharmacol. Drug. Dev. 7 (2017) 474-483. doi:10.1002/cpdd.387.
17 L.R. Webster, T. Yamada, J.C. Arjona Ferreira. A phase 2b, randomized, double-blind placebo-controlled study to evaluate the efficacy and safety of naldemedine for the treatment of opioid-induced constipation in patients with chronic noncancer pain. Pain Med. 18 (2017) 2350-2360. doi:10.1093/pm/pnw325.
